# A case report of male breast cancer in a very young patient: What is changing?

**DOI:** 10.1186/1477-7819-9-16

**Published:** 2011-02-03

**Authors:** Marcelo Madeira, André Mattar, Rodrigo José Barata Passos, Caroline Dornelles Mora, Luiz Henrique Beralde Vilar Mamede, Viviane Hatsumi Kishino, Thomas Zurga Markus Torres, Andressa Fernandes Rodrigues de Sá, Roberto Euzébio dos Santos, Luiz Henrique Gebrim

**Affiliations:** 1 Senology Discipline, São Paulo Federal University, São Paulo, Brazil; 2 Senology Discipline, UNINOVE University, São Paulo, Brazil; 3 Centro de Referência da Saúde da Mulher (CRSM), São Paulo, Brazil

## Abstract

Male breast cancer accounts for 1% of all breast cancer cases, and men tend to be diagnosed at an older age than women (mean age is about 67 years). Several risk factors have been identified, such as genetic and hormonal abnormalities.

The present study reported the case of a 25-year-old man who was diagnosed with an advanced invasive ductal carcinoma; however, he did not have any important risk factors.

Even though more data is emerging about this disease, more efforts to understand risk factors, treatment options and survival benefits are needed. In this case, we discussed the risk factors as well as the impaired fertility associated with breast cancer therapies.

## Background

Breast cancer in men is rare, and it accounts for about 1% of all malignant breast neoplasm cases [1,2]. The estimated incidence is 1 case for each 100,000 men. In the United States, about 1,910 new cases were diagnosed in 2009, and 440 of these cases resulted in death [3]. Among the histologic types, invasive ductal carcinoma is the most prevalent breast cancer in males, with an incidence varying from 65 to 95% [2,4].

Male breast cancer has unimodal age-frequency distribution with a peak incidence at 71 years old. Conversely, female breast cancer has a bimodal age-frequency distribution with early-onset and late-onset peak incidences at 52 and 72 years old, respectively [5].

This study examined a 25-year-old man without important risk factors who was diagnosed with invasive ductal carcinoma. Although it is rare, there have been instances of breast cancer in younger males [6]. We evaluated the main aspects of the epidemiology of breast neoplasm in men and the best approach for treatment.

## Case presentation

A 25-year-old Brazilian male was referred to our institution in August 2007 complaining of a breast tumor of progressive growth for the previous eight months. Previous medical and family history did not appear to contribute to the present illness. He denied using drugs or anabolic steroids and did not drink alcohol. The only medication he was taking was phenobarbital, which he had been taking for four years since he presented with two seizure episodes. The patient was a smoker who consumed 10 cigarettes per day. He also reported a normal sexual life, but he did not have children.

Physical examination revealed a 3.5 cm tumor located on the right breast. There was a retraction of the nipple; the nodule, which could be moved, had a hardened consistency and did not adhere to deep planes. The armpits did not present lymphadenopathy.

**Figure 1:**
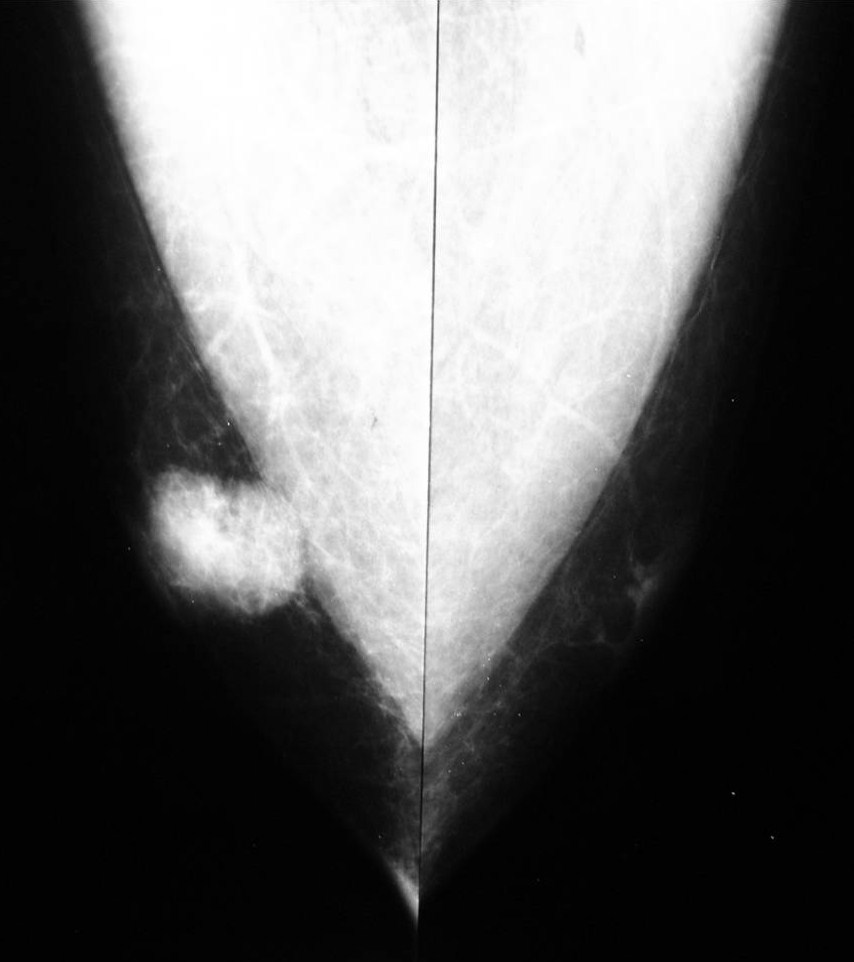
**Mammographic findings. Noncalcified high density mass of right breast**.

Mammographic findings consisted of a noncalcified high density mass (Figure 1) and breast ultrasonography revealed a hypoechogenic nodule of irregular shape with partially defined limits measuring 17 × 13 × 11 mm in the right breast. The magnetic nuclear resonance imaging showed a retroareolar nodule in the right breast, which corresponded to an expansive process. There were also signs of infiltration of the pectoralis muscle and a small area of retroareolar highlight in the left breast. Final Breast Imaging Reporting and Data System (BI-RADS) category was 5: highly suggestive of malignancy.

**Figure 2:**
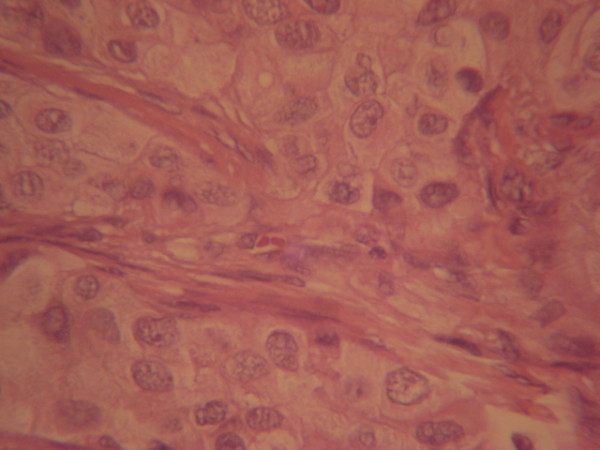
**Histological biopsy: invasive ductal carcinoma (hematoxylin-eosin staining)**.

**Figure 3:**
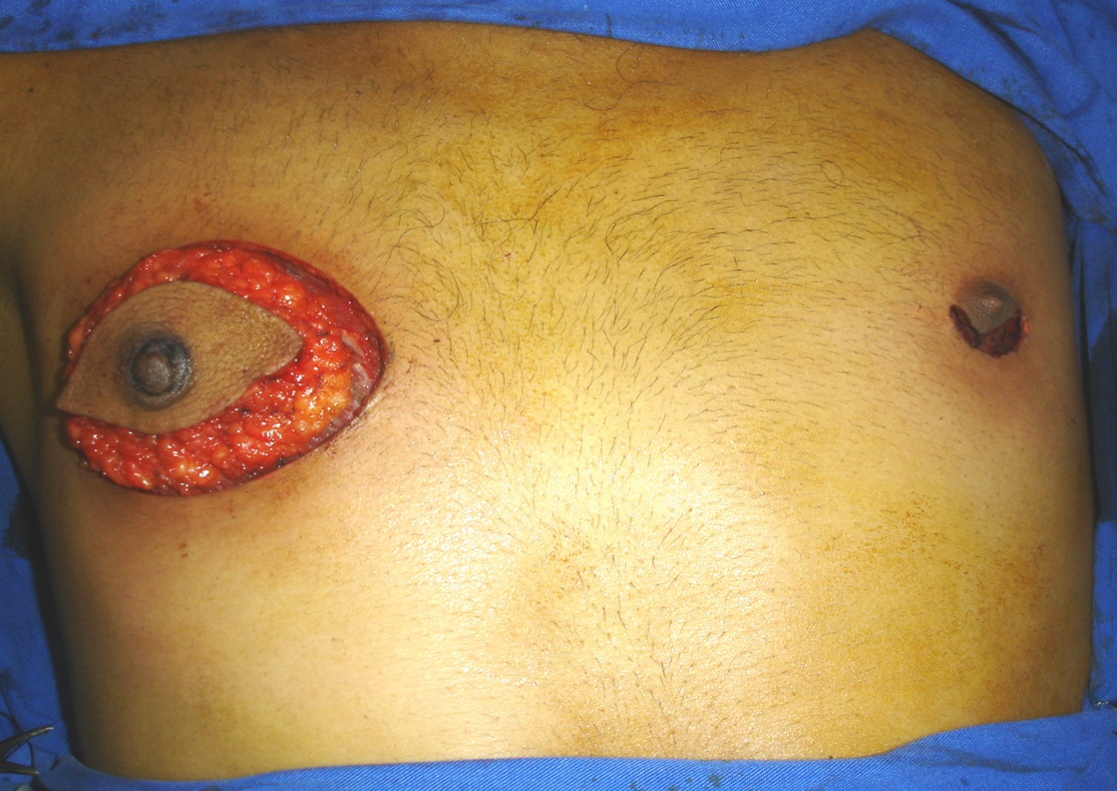
**Surgery**. Modified radical mastectomy (right breast) and retroareolar lumpectomy (left breast).

Fine-needle aspiration and a core biopsy of the lesion were performed, and the diagnosis was invasive ductal carcinoma (Figure 2). After a recommended sperm cryopreservation, the patient started neoadjuvant chemotherapy (4 × FEC 100 + 1 cisplatin 75 with adriamycin 60). In February 2008, the patient was submitted to a modified radical mastectomy (right breast) and retroareolar lumpectomy (left breast) (Figure 3).

The anatomopathological analysis confirmed the diagnosis of invasive ductal carcinoma with a 3.0-cm lesion in the biggest axle, which was histologic grade 2 and nuclear grade 2. Final breast surgical margins were free, but pectoralis muscle fascia and the nipple were infiltrated. The axillary lymph nodes dissection did not show any signs of cancer (0/8). In addition, immunohistochemical staining of the tumor was positive for estrogen and progesterone receptors, and HER-2 negative (Score 1). Although there were no signs of malignancy or atypical hyperplasia in the left breast tissue, there was fibrosclerosis and benign fibroadipose tissue.

The patient received adjuvant therapy along with radiation therapy (5,000 cGy), and tamoxifen (20 mg/day). Post-therapy follow-up were performed by members of the treatment team and included regular physical examinations and history. Liver function and alkaline phosphatase tests were not indicated during the time the patient was taking endocrine therapy. Although reports have appeared about the dangers of liver damage and hepatoma resulting from tamoxifen administration, results from NSABP studies attest such concerns have not been substantiated [7].

One year after the radiation therapy ended, the patient presented with cervical and dorsal nodules, jaundice and weight loss (about 20 kg). Evaluation of suspicious recurrent breast cancer included physical exam, the performance of a CBC, platelet count, liver function tests, chest imaging, bone scan and an abdomen ultrasound. Blood tests results were negative for hepatitis A, B and C, serum glutamic oxaloacetic transaminase 241 IU/L (normal range: 10-34), serum alanine aminotransferase 187 IU/L (7-50), lactate dehydrogenase 358 U/L (50-150), total bilirubin 8.69 mg/dl (0.3-1.9), direct bilirubin 8.40 mg/dl (0-0.3) and alkaline phosphatase 959 IU/L (20-140).

In October 2009, the abdominal ultrasonography showed the presence of several hepatic nodules. The general state of the patient was deteriorating. He had a variety of symptoms, including a lower level of consciousness, dysphagia, inappetence, fever, cyanosis, and dyspnea. The patient quickly developed multiple organ failure and died in November 2009.

Because of weakness and quick deterioration of health state of the patient, it was not possible to perform a biopsy documentation of recurrence and determination of hormone receptor status and HER-2 status.

## Discussion

Invasive ductal carcinoma in men presents peculiar features. About 42% of breast cancer cases in men are diagnosed in stage III or IV [1]. This is probably because men do not seek medical attention for breast masses as quickly as women. In addition, the tumor is usually closer to the skin in males, which increases the likelihood of infiltration into the dermis, which was reported in the present case.

Treatment strategies for male breast cancer are not based on data from randomized clinical studies in men and most treatment recommendations are extrapolated from data in women [8].

Men with breast carcinoma have a poor prognosis, especially in the younger age group, because most breast enlargements in young men are dismissed as gynecomastia [9,10]. This potential misdiagnosis can result in an unnecessary delay in treatment. The median age of breast cancer diagnosis in men is approximately 65 years old [11]. Reports of breast cancer in young male patients are rare. Nielsen and Jakobsen described a breast cancer case in a 32-year-old man [12]. More recently, an invasive cancer case was reported in a 30-year-old patient [9]. In 2008, Chang *et al. *described the case of a 16-year-old male with unilateral ductal carcinoma *in situ *and gynecomastia [13].

There is a close relation between the BRCA2 gene mutation and male breast cancer. It has also been observed, however, that some cases involve BRCA1 participation [14-16]. Other conditions that have been associated with the occurrence of breast neoplasms in men are cirrhosis [17], testicular trauma, obesity, radiation therapy exposure, and the use of exogenous estrogen [18]. In addition to the very young age of the patient in the present report, this patient did not have a family, hormonal, or genetic history that could justify the high risk for breast cancer. Although gynecomastia has been suggested to be present in 6-38% of breast cancer cases in men [19], it was not evident in our patient.

It is fundamental to consider the history of breast tumors in first-degree relatives because that can be an indicator for increased breast cancer risk. Indeed, genetic diseases such as Klinefelter's syndrome and Cowden's disease have been shown to be related to breast cancer in men [1].

There is no evidence that suggests that all men need breast magnetic nuclear resonance imaging (MRI). But suspicious MRI lesions in the contralateral breast should be examined. Furthermore, male breast cancer survivors have an increased risk of developing a second primary cancer. The risk of a contralateral breast cancer appears to be higher for men than it is for women [20]. Some studies indicate that men with breast cancer have a 30-fold increased risk of contralateral breast cancer, much greater than the two- to fourfold risk among women with breast cancer [21]. The risk of subsequent contralateral breast cancer was highest for men aged less than 50 years at the time of the first cancer diagnosis, which is consistent with studies of women with breast cancer [22,23].

Estrogen receptors and progesterone receptors have been suggested to play a role in breast cancers in men, and they are present in about 90% and 81% of breast cancers in males, respectively [4]. Furthermore, overexpression of the proto-oncogene HER-2 has been shown to present the worst prognosis for a patient [24]. Other markers that have been recently studied are p27, MIB-1 and Bcl-2 genes.

Similar to breast cancer cases in women, earlier detection of male breast cancer is correlated with the success of the treatment. Although males have considerably less mammary parenchyma than women, the investigation must be a combination of a clinical exam, mammography, cytology, and percutaneous biopsies [25,26]. The core needle biopsy is important because it enables a definitive diagnosis of invasive breast cancer and the evaluation of estrogen receptors, progesterone receptors, and Her-2 status [3].

Tamoxifen should still be considered as the optimal adjuvant therapy option for male patients with endocrine responsive disease. The effect regarding rate and overall survival by adjuvant chemotherapy is also far less well studied [8]. Some studies have demonstrated an improved disease-free and overall survival compared with historical controls using adjuvant anthracycline-based therapies [4,5,27].

Because of the high probability of an indefinite period of infertility following chemotherapy, sperm cryopreservation should be recommended for all young patients with cancer prior to the start of chemotherapy. Although treatment and survival represent the primary goals of the clinical approach towards breast cancer patients, the quality of life after treatment, including the possibility of becoming fathers, requires consideration. In addition, sperm cryopreservation is another hope that encourages young patients with cancer during and after treatment [28].

Breast cancer therapeutics in men must be based on certain parameters, such as tumor size, the presence of estrogen and progesterone receptors, HER-2 expression, and the association with other diseases. Men diagnosed with breast cancer present risk factors, such as chronic hepatopathies, that are directly associated with the neoplasm. In addition, men diagnosed with breast cancer are generally older and present other comorbidities. Due to the smaller size of male mammary parenchyma, the elected surgical treatment is modified radical mastectomy.

## Conclusions

Invasive ductal carcinoma in young men is extremely rare; the peak incidence is around the seventh decade of life. Risk factors for male breast cancer include genetic factors and hormonal abnormalities. Despite an absence of a familial history of breast cancer, hormonal abnormalities, or a genetic disease, the male patient in the present study developed breast cancer at a very young age. The causative factors in this patient were unable to be definitively identified. The pathophysiology of breast cancer in males is not adequately understood. As more cases of breast cancer in young male patients are investigated, we may be able to gain a better understanding of the mechanism.

## Competing interests

The authors declare that they have no competing interests.

## Consent

Written informed consent was obtained from the patient's family for publication of this case report and accompanying images. A copy of the written consent is available for review by the Editor-in-Chief of this journal.

## Authors' contributions

AM, RJBP, CDM and RES took part in the care of the patient. MM, LHBVM, VHK, TZMT and AFRS were responsible for the literature review, design, and writing of the manuscript. LHG was responsible for the manuscript completion and critical review. All authors read and approved the final manuscript.
